# Single-cell analysis reveals expanded CD8^+^  *GZMK*^high^ T cells in CSF and shared peripheral clones in sporadic amyotrophic lateral sclerosis

**DOI:** 10.1093/braincomms/fcae428

**Published:** 2024-11-27

**Authors:** Hyo Jae Kim, Jae-Jun Ban, Junho Kang, Hye-Ryeong Im, Sun Hi Ko, Jung-Joon Sung, Sung-Hye Park, Jong-Eun Park, Seok-Jin Choi

**Affiliations:** Graduate School of Medical Science and Engineering, Korea Advanced Institute of Science and Technology, Daejeon 34141, Republic of Korea; Department of Neurology, Seoul National University Hospital, Seoul National University College of Medicine, Seoul 03080, Republic of Korea; Graduate School of Medical Science and Engineering, Korea Advanced Institute of Science and Technology, Daejeon 34141, Republic of Korea; Department of Neurology, Seoul National University Hospital, Seoul National University College of Medicine, Seoul 03080, Republic of Korea; Department of Neurology, Seoul National University Hospital, Seoul National University College of Medicine, Seoul 03080, Republic of Korea; Department of Neurology, Seoul National University Hospital, Seoul National University College of Medicine, Seoul 03080, Republic of Korea; Department of Pathology, Seoul National University Hospital, Seoul National University College of Medicine, Seoul 03080, Republic of Korea; Graduate School of Medical Science and Engineering, Korea Advanced Institute of Science and Technology, Daejeon 34141, Republic of Korea; Department of Neurology, Seoul National University Hospital, Seoul National University College of Medicine, Seoul 03080, Republic of Korea

**Keywords:** amyotrophic lateral sclerosis, cerebrospinal fluid, single-cell RNA sequencing, T-cell receptor, clonality

## Abstract

Amyotrophic lateral sclerosis (ALS) is a fatal neurodegenerative disease that affects motor neurons in the brain and spinal cord. Despite the crucial role of aberrant immune responses in ALS pathogenesis, studies investigating immunological profiles in the cerebrospinal fluid (CSF) of patients with ALS have reported inconsistent findings. Herein, we explored the intrathecal adaptive immune response and features of circulating T cells between CSF and blood of patients with ALS using single-cell RNA and T-cell receptor (TCR) sequencing. This study comprised a total of 11 patients with apparently sporadic ALS and three controls with non-inflammatory diseases. We collected CSF from all participants, and for three patients with ALS, we additionally obtained paired samples of peripheral blood mononuclear cells (PBMCs). Utilizing droplet-based single-cell RNA and TCR sequencing, we analysed immunological profiles, gene expression characteristics and clonality. Furthermore, we examined T-cell characteristics in both PBMC and CSF samples, evaluating the shared T-cell clones across these compartments. In the CSF, patients with ALS exhibited a lower proportion of CD4^+^ T cells (45.2 versus 61.2%, *P* = 0.005) and a higher proportion of CD8^+^  *GZMK*^hi^ effector memory T cells (TEMs) than controls (21.7 versus 16.8%, *P* = 0.060). Higher clonality was observed in CD8^+^ TEMs in patients with ALS compared with controls. In addition, CSF macrophages of patients with ALS exhibited a significant increase in chemokines recruiting CD8^+^ TEMs. Immunohistochemical analysis showed slightly higher proportions of T cells in the perivascular and parenchymal spaces in patients with ALS than in controls, and CD8^+^ TEMs co-localized with neurons or astrocytes in the motor cortices of patients with ALS. Clonally expanded CD8^+^  *GZMK*^hi^ TEMs primarily comprised shared T-cell clones between CSF and PBMCs. Moreover, the shared CD8^+^ TEMs of PBMCs exhibited gene expression profiles similar to CSF T cells. Patients with ALS showed an increase in proportion and clonality of CD8^+^  *GZMK*^hi^ TEMs and activated features of macrophages in CSF. The shared T-cell clone between CSF and blood was mainly composed of expanded CD8^+^  *GZMK*^hi^ TEMs. In conclusion, single-cell immune profiling provided novel insights into the pathogenesis of ALS, characterized by activated macrophages and clonally expanded CD8^+^ T cells potentially communicating with the central nervous system and peripheral circulation.

## Teaser

Kim *et al*. report clonally expanded CD8^+^  *GZMK*^high^ T cells in the CSF of patients with sporadic amyotrophic lateral sclerosis (ALS). They demonstrate that these cells share expanded clones with those in the blood and co-localize with motor neurons, suggesting a pathogenic role for cytotoxic CD8^+^ T cells in ALS.

## Graphical Abstract

**Graphical Abstract:**
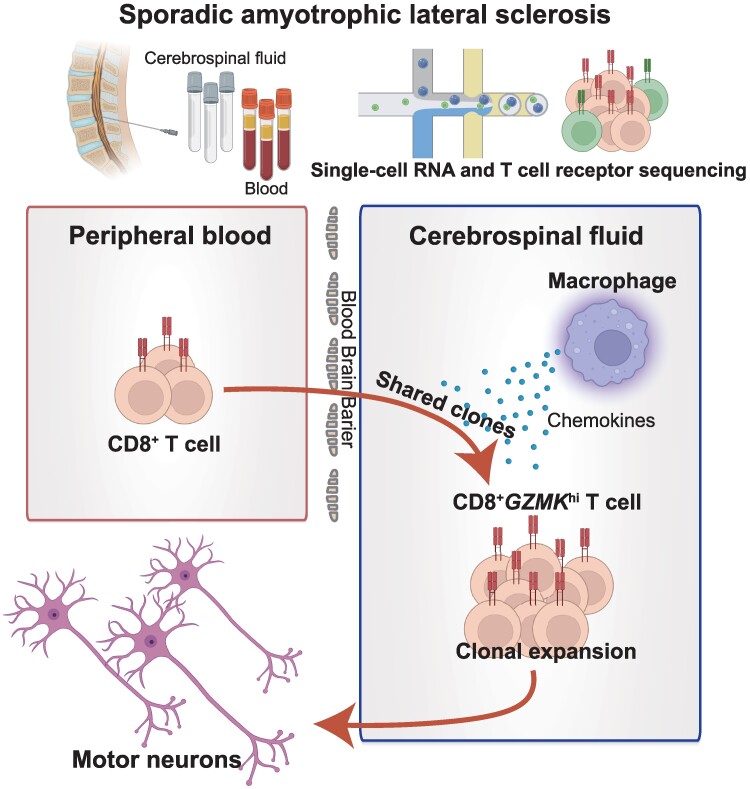

## Introduction

Amyotrophic lateral sclerosis (ALS) is a fatal neurodegenerative disease marked by the progressive degeneration of motor neurons in the brain and spinal cord, culminating in death due to respiratory failure.^1^ ALS is characterized as a non-cell autonomous disease where central nervous system (CNS) glial cells, such as microglia and astrocytes, are involved in motor neuron degeneration.^2,3^ Although debate surrounds the pathogenesis and progression of ALS, a growing body of evidence points to a dysregulated immune response as an important factor contributing to the heterogeneity of ALS.^4,5^ Furthermore, peripheral immune activation plays a significant role in the pathogenesis of ALS, highlighting the critical interplay between the peripheral and central immune systems.^6-9^

Consistent with preclinical studies highlighting the importance of immune cells in ALS pathogenesis, the passive transfer of regulatory T cells (Tregs) into ALS mice markedly slowed disease progression and prolonged survival.^10^ Recent research observed a shift of the peripheral immune profile towards a CD4^+^ type 1 and type 17 T helper cell-mediated proinflammatory response, but no significant changes were noted in the cerebrospinal fluid (CSF) immune cells, except for a decrease in the percentage of CD8^+^ naïve T cells.^11^ The role of CD8^+^ T cells in ALS pathogenesis remains underexplored compared with CD4^+^ T cells. Self-reactive CD8^+^ T cells in *SOD1* mutant rodents prompted selective motor neuronal death,^12^ and clonal expansion of CD8^+^ T cells in peripheral blood was observed in patients with ALS-associated *SETX* mutation.^13^ The role of cytotoxic T cells in sporadic ALS, particularly within the CSF, however, remains poorly understood. Furthermore, the significance of tissue-resident macrophages in the CNS [microglia- and border-associated macrophages (BAMs)] in the pathogenesis of neurodegeneration is also increasingly recognized.^2,14^ However, the features of these yolk-sac-derived macrophages in the CSF remain poorly characterized.

Although both upper and lower motor neurons progressively degenerate in ALS, prior analyses have predominantly focused on peripheral immune profiling,^15-17^ thus providing a limited perspective on the CSF.^11^ To shed light on the immunopathogenesis of ALS within the CNS compartment, we examined the immune landscape of CSF and peripheral blood mononuclear cells (PBMCs) from patients with ALS and controls through transcriptomic and T-cell receptor (TCR) analyses at single-cell resolution. Moreover, we confirmed the infiltration of CD8^+^ T cells in post-mortem brain tissues from patients with ALS with immunohistochemical analysis.

## Materials and methods

### Study population and sample preparation

Patients with sporadic ALS were recruited from ALS clinics at Seoul National University Hospital and Inha University Hospital between June 2020 and May 2022. The eligibility criteria included the absence of first-degree relatives with ALS, frontotemporal dementia or psychiatric disorders and a diagnosis of definite, probable or possible ALS according to the revised El Escorial criteria.^18^ Patients concurrently diagnosed with systemic infections, autoimmune diseases or cancers were excluded, as were those taking steroids. ALS-associated genes, including *SOD1, FUS, TARDBP* and *SETX,* were examined using targeted gene panels or whole-genome sequencing. As *C9orf72* hexanucleotide repeat expansion has not been identified in the Korean population,^19^ it was not tested in our patients. In patients with ALS, the ALS Functional Rating Scale—Revised score and King’s clinical stage were examined. Non-inflammatory controls, free of signs of infection or inflammation, were included if they had undergone a spinal puncture for diagnostic purposes. Supplementary Table 1 provides detailed clinical information for both patients with ALS and controls. This study was approved by the ethics committees of Seoul National University Hospital (IRB no. 1904-165-1031) and Inha University Hospital (IRB no. 2018-08-009). Written informed consent was obtained from all participants in accordance with the Declaration of Helsinki.

We included CSF samples with a red blood cell count of <5/mm^3^ from all participants for the investigation of immunological profiles restricted to the CNS compartment. For singleplex samples, 10–15 cc of CSF was collected within 2 h of the spinal puncture and then centrifuged at 500× *g* for 5 min. The cell-free supernatant was discarded, and the cell pellet was resuspended in cold 1× phosphate-buffered saline (PBS) with 0.04% bovine serum albumin. For multiplexed samples, we thawed three frozen CSF stocks stored in liquid nitrogen in a 37°C water bath, combined them into a single tube, added RPMI1640 with 10% fetal bovine serum and then centrifuged the mixture at 700× *g* for 5 min. The cell-free supernatant was discarded, and the cell pellet was resuspended in cold 1× PBS with 0.04% bovine serum albumin. Genomic DNA for demultiplexing was extracted from white blood cells paired with the CSF samples. PBMCs were isolated from peripheral venous blood within 6 h using Ficoll-Paque density gradient centrifugation and stored in liquid nitrogen. Multiplexed PBMC samples were prepared and demultiplexed in the same manner as described above.

### Single-cell RNA and TCR library preparation and sequencing

Droplet-based single-cell 5′ and V(D)J Reagent Kits (10× Genomics) were utilized to generate single-cell cDNA libraries following the protocol of the Chromium Single-Cell Immune Profiling Solution (10× Genomics). Libraries of cDNA were pooled and sequenced throughout multiple lanes of NovaSeq 6000 (Illumina) to achieve a read depth of over 50 000 reads per cell.

### Single-cell RNA sequencing data processing and analysis

Raw single-cell transcriptomic data were aligned using Cell Ranger (version 6.0.1, 10× Genomics) with the GRCh38 human reference genome (version 2020-A). Cells with fewer than 500 detected genes and 2000 UMI counts were considered empty droplets and subsequently removed. Cells with more than 7000 detected genes were considered potential doublets and removed from analysis. The Scanpy (version 1.8.2) Python package was used to load the cell-gene count matrix and analysis.^20^ Scrublet was used for doublet detection.^21^ For multiplexed samples, Souporcell was used for demultiplexing.^22^ Subsequently, filtered count matrices underwent count-normalization and log-transformation and were processed through a series of steps. Highly variable genes were identified using the default settings in Scanpy. Following this, the expression matrices were scaled, and principal component analysis was conducted, specifying 50 principal components. To correct for batch effect specific to each patient, the principal components were adjusted using Harmony.^23^ The uniform manifold approximation and projection (UMAP) coordinates were then determined by utilizing the neighbourhood graphs. For the initial clustering of cells, the Leiden algorithm was employed.^24^

### TCR repertoire analysis

We aligned the single-cell paired alpha and beta chains of the raw TCR sequencing data set using CellRanger-vdj (v.6.0.1, 10× Genomics), with subsequent data analysis performed with Scirpy (v.0.10.1).^25^ We discarded low-quality TCRs, such as those presenting multiple pairs of α and β chains or single α/β chains. We defined clones as TCRs having the same V(D)J segment and identical CDR3 nucleotide sequence in both α and β chains. We categorized the number of clones (count of T cells with the same TCR) into distinct groups: unique, 2–4, 5–8 and over 8. Subsequently, we computed and compared the proportion of clonal T cells for each T-cell subtype to assess clonality. To quantify the diversity of TCR repertoires, we calculated the normalized Shannon entropy for each T-cell subset using Scirpy.

### Immunohistochemistry

We prepared 5-µm-thick spinal cord and brain sections from formalin-fixed paraffin-embedded tissue blocks, which underwent deparaffinization in xylene. These sections were incubated with the following primary antibodies: CD4 (VENTANA, 790-4423) and CD8 (Ventana, 790-4460). Primary antibody reactions were visualized using an OptiView universal DAB (3,3′-diaminobenzidine) staining kit (Ventana, 760-700) and counterstained with haematoxylin. We quantified perivascular infiltration of T cells by counting the number of blood vessels with stained T cells in the perivascular space and dividing it by the total number of blood vessels present on the slide. To quantify the parenchymal infiltration of T cells, we counted the number of stained cells and divided it by the total area (mm^2^) of the image. A total of seven images from the spinal cord, motor cortex and cerebellum [three from patients with ALS-Frontotemporal lobar degeneration (ALS-FTLD) and four from controls] were captured using the Aperio-AT2 slide scanner (Leica Biosystems). All counting procedures were performed manually by an examiner who was blinded to the sample identities.

For immunofluorescence staining, tissue sections were first deparaffinized with graded ethanol, followed by immersion in Tris-buffered saline. The sections were then incubated with TrueBlack Autofluorescence Quencher (Biotium) and Protein Block solution (Vector Labs). Primary antibodies against CD8 (Thermo Fisher, MA5-14548, 1:100), CD45RA (Thermo Fisher, 14-0458-82, 1:100), CCR7 (Thermo Fisher, 14-1979-82, 1:100), MAP2 (Millipore, AB-5622, 1:200) and GFAP (Thermo Fisher, PA1-10004, 1:200) were applied to the tissue slides and incubated for 15 h at room temperature. After washing with PBS, the sections were incubated with secondary antibodies, including Alexa Fluor 488 goat anti-mouse IgG (Thermo Fisher, A-11001, 1:300), Alexa Fluor 568 goat anti-rabbit (Thermo Fisher, A-11011, 1:300) and Alexa Fluor 647 donkey anti-rat (Thermo Fisher, A-48272, 1:300) for 1 h at room temperature. The stained sections were then mounted with Prolong Gold Antifade with DAPI (Thermo Fisher) for nuclear counterstaining. Images were captured using an Olympus FV3000 microscope with Fluoview FV31S software (Olympus). The clinical information pertaining to the analysed autopsy tissues is presented in Supplementary Table 2.

### Statistical analysis

The Mann–Whitney U-test was utilized for comparing the proportions of cell subtypes. The χ^2^ test was employed to compare the proportion of clonal T cells of each T-cell subtype between groups. The Spearman correlation test was utilized to analyse the correlation between cell proportion and clinical severity. The Kruskal–Wallis test was performed for non-parametric analyses among three groups. For analyses according to King’s stages, ordinal logistic regression analysis was employed. In differentially expressed gene (DEG) analysis, a pseudo-bulk matrix was constructed by aggregating counts across samples and cell types. Subsequently, a general linear regression model in pyDESEQ2 was utilized to calculate *P*-values.^26^ When creating the pseudo-bulk matrix, individuals with fewer than 10 cells were excluded from the DEG analysis.

## Results

### Single-cell RNA sequencing of CSF in patients with sporadic ALS and controls

**Figure 1:**
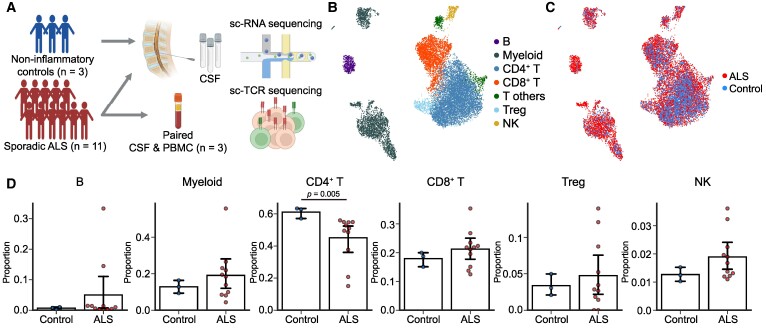
**Single-cell landscape of CSF immune cells.** (**A**) Study scheme for single-cell RNA and TCR sequencing of CSF samples from 11 patients with sporadic ALS (with three additional paired PBMCs) and three controls without inflammatory diseases. Created in BioRender (2024). https://BioRender.com/v57g779. (**B, C**) UMAP visualization of CSF cells in patients with ALS and controls without inflammatory diseases coloured by cell type (**B**) and disease group (**C**). (**D**) Bar plot depicting CSF cell type proportions between ALS and controls. *P*-values were calculated using the Mann–Whitney U-test.

The study enrolled 11 patients diagnosed with evidently sporadic ALS, along with three controls who did not have inflammatory diseases. We conducted single-cell RNA analyses of CSF immune cells as well as TCR analyses of T cells (Fig. 1A). From the CSF, we obtained 13 223 single-cell RNA and 9959 single-cell TCR profiles. We employed unsupervised graph-based clustering and visualizations through UMAP for the CSF immune cells (Fig. 1B and C). Then, we annotated the cell types manually based on their respective marker gene expression profiles. Upon comparing the distribution of immune cells categorized by general lineage, the proportion of CD4^+^ T cells was lower in patients with ALS than in the control group (45.2 versus 61.2%, *P* = 0.005), while the remaining cell types showed no significant differences (Fig. 1D).

### Differences in T-cell features and clonal expansion in the CSF of patients with ALS

**Figure 2:**
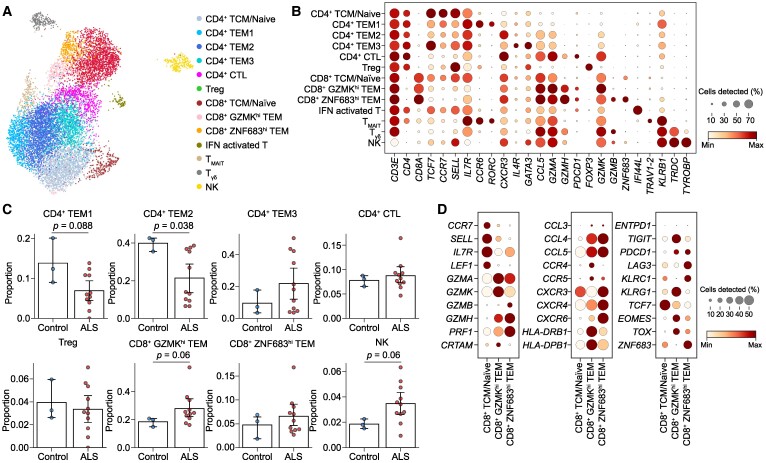
**T-cell and NK cell characteristics in the CSF and comparison of proportion between patients with ALS and controls without inflammatory diseases.** (**A**) UMAP visualization of the subclustering of T and NK cells in the CSF coloured by cell type. (**B**) Dot plot of marker gene expression in T-cell and NK cell subtypes. The size of the dots denotes the proportion of cells expressing marker genes, and the colour represents the min–max normalized expression of marker genes in each group. (**C**) Bar plot depicting T-cell and NK cell subtype proportions between ALS (*n* = 11) and controls (*n* = 3). *P*-values were calculated using the Mann–Whitney U-test. (**D**) Dot plot of marker gene expressions related to T-cell functions in each CD8^+^ T-cell subtype. The size of the dots denotes the proportion of cells expressing the genes, and the colour represents the min–max normalized expression of genes in each group. TCM, central memory T cell; IFN, interferon.

CD4^+^ T cells were categorized as CD4^+^ central memory/naïve T cells (CD4^+^ central memory T cell/naïve; expressing markers *CCR7* and *SELL*), CD4^+^ TEMs, type 1 (CD4^+^ TEM1; *CCR6* and *RORC*), CD4^+^ TEM2 (*CXCR3*), CD4^+^ TEM3 (*IL4R* and *GATA3*), CD4^+^ cytotoxic T cells [CD4^+^ cytotoxic T lymphocyte (CTL); *GZMH* and *PDCD1*] and CD4^+^ regulatory T cells (Treg; *FOXP3*). CD4^+^ TEM1 demonstrated Th17-like characteristics, while CD4^+^ TEM3 appeared to exhibit Th2-like properties. CD8^+^ T cells were subdivided into the following subsets: CD8^+^ central memory T cell/naïve expressing naive markers (*CCR7*) and two distinct CD8^+^ TEM clusters (CD8^+^  *ZNF683*^hi^ TEM and CD8^+^  *GZMK*^hi^ TEM). Details regarding the annotations and marker genes for these subpopulations are summarized in Fig. 2A and B.

Following cell type annotations, we compared the proportion of T-cell populations in the CSF between the patients with ALS and controls. The proportion of CD8^+^  *GZMK*^hi^ TEMs (21.7 versus 16.8%, *P* = 0.060) was higher in the ALS group than in the control group (Fig. 2C). The proportion of natural killer (NK) cells also tended to be higher in the patients with ALS than in controls. However, no significant difference was found in the proportion of Tregs between the two groups. Two patients (ALS8 and ALS11) showed extremely rapid progression (Supplemental Table 1). Thus, we conducted an additional analysis excluding these patients and found that CD8^+^  *GZMK*^hi^ TEMs remained significantly more abundant in the ALS group (19.4 versus 13.6%, *P* = 0.036). Due to the limited size of our control group, we incorporated additional data from a public single-cell RNA sequencing data set (GSE133028), which includes CSF samples from three controls and patients with multiple sclerosis for further comparison (Supplementary Fig. 1). The proportion of CD8^+^  *GZMK*^hi^ TEMs tended to be higher in ALS compared with the expanded control group (Kruskal–Wallis test *P* = 0.093, adjusted *P* = 0.094) and was similar to the levels observed in patients with multiple sclerosis (Supplementary Fig. 1B). Our data set was obtained from either frozen or fresh CSF samples; however, no significant differences in T-cell subset compositions were observed between these sample types, except for CD4^+^ TEM3 (Supplementary Fig. 1C).

**Figure 3:**
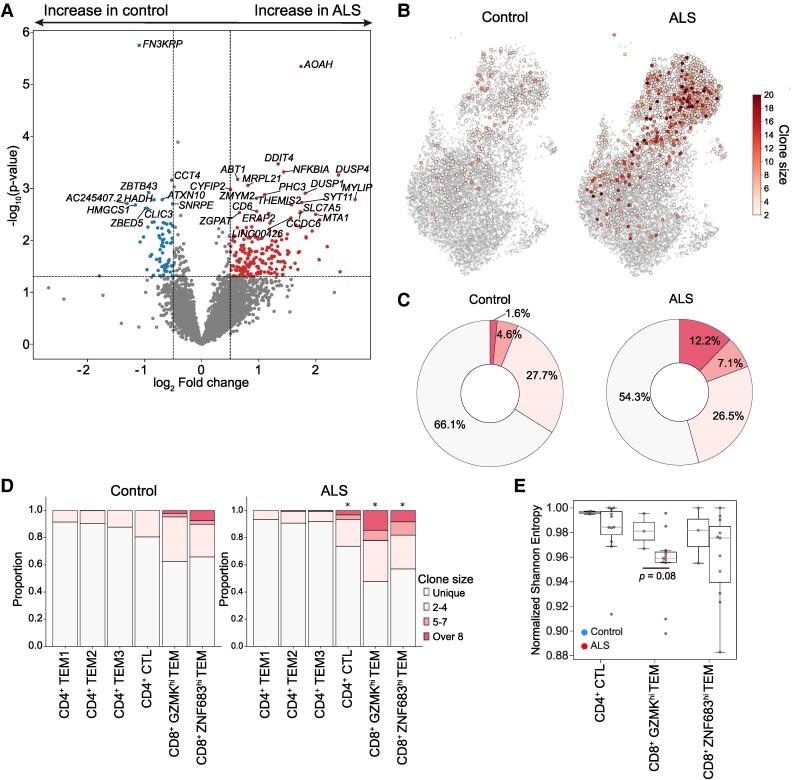
**Comparison of T-cell features and clonality in the CSF between patients with ALS and controls without inflammatory diseases.** (**A**) Volcano plot showing DEGs between ALS and controls in CD8^+^  *GZMK*^hi^ TEMs. *P*-values were calculated using pyeDESeq2’s Wald test on negative binomial generalized linear models, and fold changes are presented as log_2_-transformed normalized count ratios. (**B**) UMAP visualization of clonal clusters of T cells. Unique T cells were plotted as single grey dots and clonal T cells as circles. The colour indicates the clone size of each T-cell clone. (**C**) Pie chart showing the proportions of the clonally expanded CD8^+^ T-cell population in ALS (*n* = 11) and controls (*n* = 3). (**D**) Bar plot illustrating the clonally expanded T-cell proportion for each subtype in ALS (*n* = 11) and controls (*n* = 3). The χ² test was used for statistical comparisons of proportions. Statistically significant results from the χ² tests are presented for specific cell types: CD4^+^ CTL (χ² = 7.0, *P* = 0.008), CD8^+^  *GZMK*^hi^ TEM (χ² = 21.1, *P* < 0.001) and CD8^+^  *ZNF683*^hi^ TEM (χ² = 7.4, *P* = 0.006). (**E**) Boxplot depicting T-cell diversity of CD4^+^ CTL, CD8^+^  *GZMK*^hi^ TEM and *ZNF683*^hi^ TEM populations as measured by normalized Shannon entropy in ALS (*n* = 11) and controls (*n* = 3). IFN, interferon.

The CD8^+^  *GZMK*^hi^ TEMs exhibited increased *TOX* and *TIGIT* expression and reduced expression of *TCF7* (Fig. 2D), aligning with the well-known traits of CD8^+^ terminally differentiated effector memory T cells (TEMRAs).^13^ Moreover, *HLA-DPB1/DRB1, CRTAM* and *EOMES* were upregulated in these subsets, indicating T-cell activation.^27^ Both CD8^+^ TEM subpopulations displayed elevated expression of cytotoxic molecules; however, in contrast to the CD8^+^  *GZMK*^hi^ TEMs, the CD8^+^  *ZNF683*^hi^ TEMs exhibited high expression of *GZMB* (Fig. 2D). In the DEG analysis, CD8^+^  *GZMK*^hi^ TEMs from patients with ALS showed increased expression of several genes compared with the control group. These included *AOAH* (a gene that encodes a lipase known as acyloxyacyl hydrolase), *DUSP1* and *DUSP4* (associated with T-cell differentiation and activation), *CD6* (a gene that encodes a pivotal adhesion molecule in TCR-related T-cell activation) and *STAT5B* (Fig. 3A and Supplementary Fig. 2). No CD4^+^ T-cell subsets were enriched in patients with ALS. Conversely, the proportions of both CD4^+^ TEM1 (*CCR6* and *RORC* high) and CD4^+^ TEM2 (*CXCR3* high) were elevated in controls compared with patients with ALS. DEG analysis of CD4^+^ TEMs yielded no significant findings (Supplementary Fig. 2).

Next, we assessed the clonality of T cells in CSF using single-cell TCR sequencing. We defined a clone as possessing identical V and J regions, along with CDR3 sequences in both alpha and beta chains. Clonal T-cell expansion was observed to a greater extent in patients with ALS than in controls (Fig. 3B), and most of the large clones were located within the CD8^+^  *GZMK*^hi^ TEM cluster. The proportion of expanded clones (clonal size ≥ 8) in CD8^+^ T cells was over seven times higher in patients with ALS than in controls (12.2 versus 1.6%; Fig. 3C). Within each T-cell subcluster, the clonal proportions of CD8^+^  *GZMK*^hi^ TEMs, CD8^+^  *ZNF683*^hi^ TEMs and CD4^+^ CTLs in patients with ALS were significantly higher than in controls (Fig. 3D). TCR diversity analysis (Fig. 3E) using normalized Shannon entropy revealed a trend towards lower diversity (indicating higher clonality) in CD8^+^  *GZMK*^hi^ TEMs in patients with ALS compared with controls (*P* = 0.088). In contrast, CD8^+^  *ZNF683*^hi^ TEMs and CD4^+^ CTLs showed no significant differences in TCR diversity between ALS and control groups. By comparing the CDR3 motif of T cells to a public antigen-specific motif database (vdjdb),^28^ we detected the presence of virus-associated motifs in some T-cell clones. While several T-cell clones related to cytomegalovirus were identified, most expanded clones did not exhibit this association (Supplementary Fig. 3).

Furthermore, we assessed the relationships between T-cell properties and the clinical characteristics of ALS (Supplementary Fig. 4). A significant positive correlation was observed within T cells between the proportion of CD4^+^ T cells and ALS Functional Rating Scale—Revised (*r* = 0.839, *P* = 0.001), whereas a significant negative correlation was identified between the proportion of CD8^+^  *GZMK*^hi^ TEMs and ALS Functional Rating Scale–Revised (*r* = −0.702, *P* = 0.016; Supplementary Fig. 4A and B). However, when excluding ALS8 and ALS11, who exhibited markedly rapid progression, both correlations lost statistical significance. Upon analysing the proportions of T-cell subclusters in patients with ALS according to their clinical stage (Supplementary Fig. 4C and D), we noted an increase in the proportion of CD8^+^  *GZMK*^hi^ TEMs as the clinical stage advanced. Similarly, the clonality of CD8^+^ T cells exhibited an increasing trend as the clinical stage advanced. Regarding age-associated immune alterations (Supplementary Fig. 4E and F), within the ALS group, we found no significant correlations between age and either the proportion of CD8^+^  *GZMK*^hi^ TEMs (*r* = 0.5, *P* = 0.101) or the clonality of CD8+ T cells (*r* = 0.197, *P* = 0.562). Lastly, among the 11 patients with sporadic ALS included in this study, three patients had a variant of uncertain significance in the *NEFH* gene. No significant differences were observed in the proportions of T-cell subclusters between patients with ALS with a variant of uncertain significance in *NEFH* and those without (Supplementary Fig. 5A). However, the clonality of CD8^+^ T cells tended to be higher in patients with ALS without a variant of uncertain significance in the *NEFH* gene, although this difference was not statistically significant (Supplementary Fig. 5B).

### Proinflammatory signature of CSF macrophages in ALS

**Figure 4:**
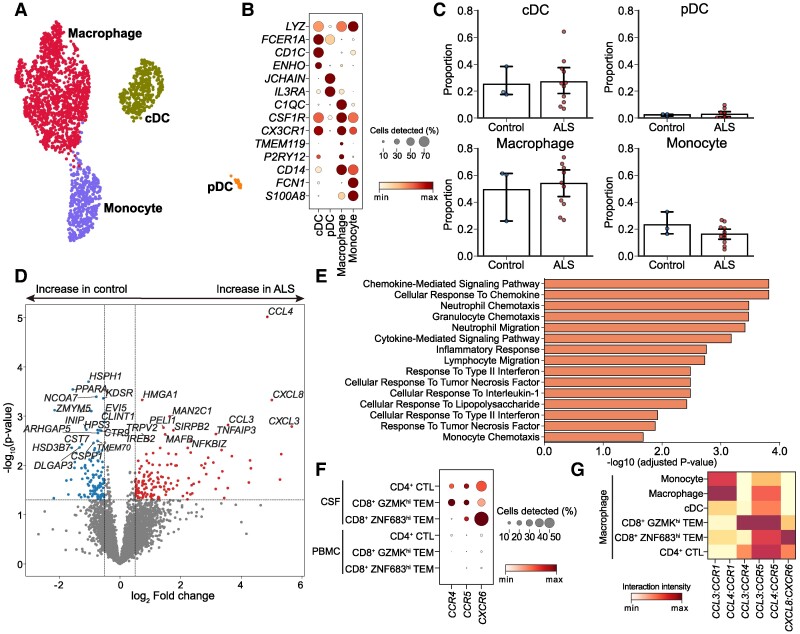
**Proinflammatory role of CSF macrophages in ALS.** (**A**) UMAP visualization of the subclustering of myeloid cells in the CSF coloured by cell type. (**B**) Dot plot of marker gene expressions in myeloid cell subtypes. The size of the dots denotes the proportion of cells expressing marker genes, and the colour represents the min–max normalized expression of marker genes in each group. (**C**) Bar plot depicting myeloid cell subtype proportions in ALS (*n* = 11) and controls (*n* = 3). Each dot represents an individual sample’s proportion within the respective group. The bars indicate the mean proportion for each group, and error bars represent the standard error of the mean. *P*-values were calculated using the Mann–Whitney U-test. (**D**) Volcano plot showing DEGs between ALS (*n* = 11) and controls (*n* = 3) in macrophages. *P*-values were calculated using a generalized linear model implemented in pyDESeq2. (**E**) Gene ontology analysis of macrophage gene signatures upregulated in patients with ALS. The significance of enrichment was determined using Fisher’s exact test, which assesses the overlap between the input gene list and each gene set. (**F**) Dot plot of chemokine gene expressions in cytotoxic T-cell subtypes in CSF and PBMCs. The size of the dots denotes the proportion of cells expressing marker genes, and the colour represents the min–max normalized expression of marker genes in each group. (**G**) Ligand–receptor analysis of macrophages with other cell types in the CSF of patients with ALS. The interaction intensity was calculated by multiplying the normalized expression values of ligand and receptors in each cell–cell pair. cDC, conventional dendritic cell; pDC, plasmacytoid dendritic cell.

Myeloid cells were categorized as conventional dendritic cells (expressing the markers *FCER1A* and *CD1C*), plasmacytoid dendritic cells (*JCHAIN* and *IL3RA*), macrophages (*C1QC*, *CSF1R*, *LYVE1*, *TMEM119* and *P2RY12*) and monocytes (*CD14* and *FCN1*; Fig. 4A and B). No significant differences in the proportions of myeloid subsets were observed between the ALS and control groups (Fig. 4C). However, DEG analysis revealed higher expression levels of cytokines such as *CCL3*, *CCL4*, *CXCL3* and *CXCL8* in the macrophages of patients with ALS compared with the control group (Fig. 4D). Gene ontology analysis using the upregulated genes in the macrophages of patients with ALS revealed significant enrichment in pathways involving chemokine-related signalling, chemotaxis in various cell types and inflammatory signalling (Fig. 4E). Importantly, the expression of *CCR4*, *CCR5* and *CXCR6*, which encode receptors for the protein products of the genes upregulated in macrophages (*CCL3*, *CCL4*, and *CXCL8*), was specifically observed in the CSF of cytotoxic T cells (Fig. 4F). This implies the acquisition of chemotactic and activated status in the cytotoxic T cells within the CSF of patients with ALS.^29^ To investigate potential cellular interactions between CSF macrophages in patients with ALS with other cell types, we focused on gene expression programmes specific to CSF macrophages of patients with ALS, including *CCL3*, *CCL4* and *CXCL8*, along with their corresponding receptor pairs. The interaction intensity was calculated by multiplying the normalized expression values of ligands and receptors within each pair of interacting cells. Ligand–receptor interaction analysis revealed strong interactions between macrophages (*CCL3* and *CCL4*) and CD8 *GZMK*^hi^ TEMs (*CCR4* and *CCR5*) within the CSF, indicating proinflammatory crosstalk between macrophages and cytotoxic T cells that may contribute to the initiation of inflammation in the CNS of patients with ALS (Fig. 4G).

### CNS infiltration of CD8^+^ terminally differentiated T cells

**Figure 5:**
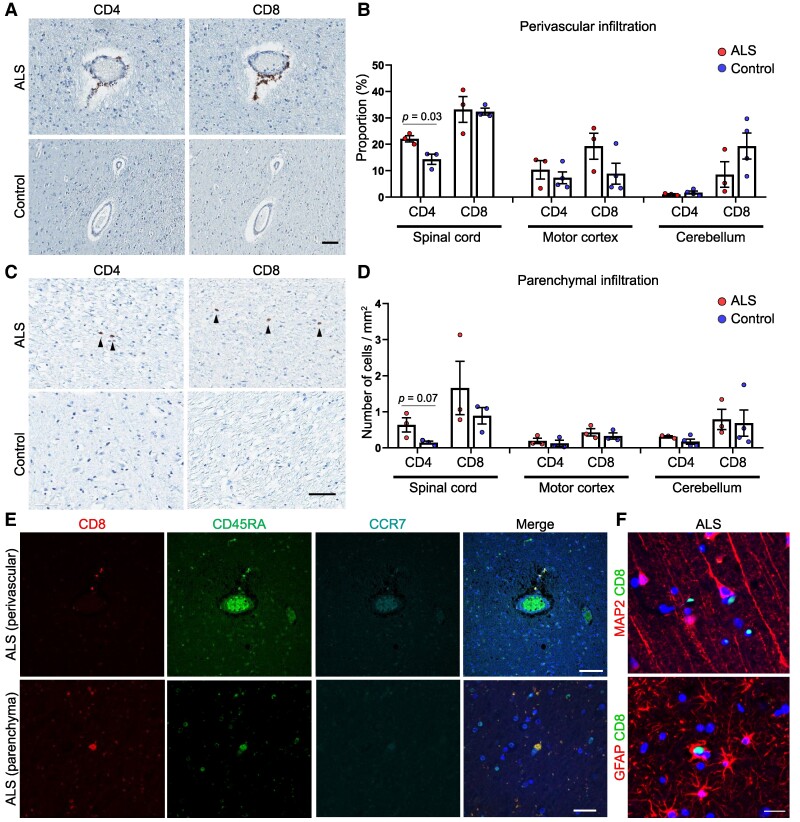
**Identification of T cells in the perivascular space and parenchyma of samples from ALS autopsies.** (**A**) Representative 3,3′-diaminobenzidine staining images of CD4^+^ and CD8^+^ T cells in the perivascular space of motor cortices from ALS-FTLD and control subjects. (**B**) Quantification of perivascular T-cell infiltration in the spinal cord, motor cortex and cerebellum of ALS-FTLD (*n* = 3) and control (*n* = 4) groups. Data are presented as mean ± SEM. Statistical comparisons between the two groups were performed using the Student’s *t*-test. The proportion of vessels with perivascular CD4^+^ T cells in the ALS spinal cord was significantly higher than in the controls (*P* = 0.03); other differences were not significant. (**C**) Representative 3,3′-diaminobenzidine staining images showing parenchymal CD4^+^ and CD8^+^ T cells (black arrows) in the spinal cord of ALS-FTLD and control subjects. (**D**) Quantification of the number of infiltrating parenchymal T cells per unit area (mm^2^) in the spinal cord, motor cortex or cerebellum of ALS-FTLD (*n* = 3) and control (*n* = 4) groups. Data are presented as mean ± SEM. Statistical comparisons between the two groups were performed using the Student’s *t*-test; the differences were not statistically significant. (**E**) Representative confocal images of infiltrating CD8^+^ CD45RA^+^ CCR7^−^ T cells in the perivascular space (upper, scale bar = 100 μm) and within the parenchyma of the motor cortex in ALS-FTLD subjects (lower, scale bar = 30 μm). (**F**) Confocal images depicting direct contact between CD8^+^ T cells and neurons (MAP2^+^, left) or astrocytes (GFAP^+^, right) in the motor cortex of ALS-FTLD subjects. Scale bar = 20 µm.

To validate the presence of clonally expanded T cells in the CSF, we investigated T-cell populations in autopsy tissues (primary motor cortices, spinal cord, and cerebellum). 3,3′-Diaminobenzidine staining revealed a slightly higher frequency of CD4^+^ and CD8^+^ T cells in both the perivascular (Fig. 5A and B) and parenchymal spaces (Fig. 5C and D) in subjects with ALS-FTLD compared with controls. Interestingly, this trend was observed exclusively in the spinal cord and motor cortices, but not in the cerebellum. Furthermore, we aimed to identify CNS-infiltrating CD8^+^ TEMRAs (CD45RA^+^ and CCR7^−^), which exhibit similar gene expression profiles to CD8^+^  *GZMK*^hi^ TEMs. This allowed us to confirm the presence of CD8^+^ TEMRAs infiltrating both the perivascular space and parenchyma (Fig. 5E), found in close proximity to neurons or astrocytes (Fig. 5F). These observations, along with our single-cell transcriptome and TCR repertoire analyses, provide evidence suggesting that clonally expanded CD8^+^ T cells in patients with ALS infiltrate the CNS and may directly assault neuronal cells, including motor neurons and astrocytes.

### Shared T-cell clones between CSF and PBMCs

**Figure 6:**
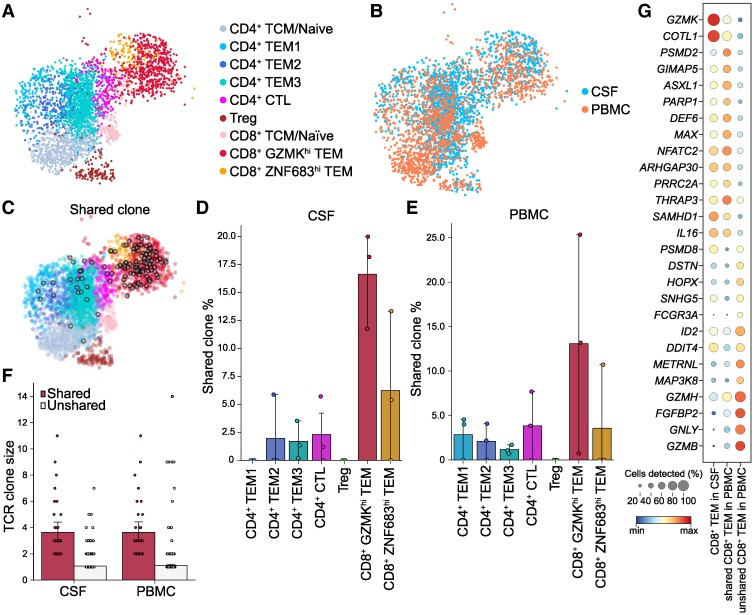
**Shared T-cell clones between CSF and blood samples in patients with ALS.** UMAP visualization of T-cell subclusters in patients with ALS, coloured by cell type (**A**) and the site of sample collection (**B**). (**C**) UMAP visualization of T-cell subclusters overlaid with shared clones between CSF and PBMCs shown in grey circles. Bar plot showing the proportion of shared and unshared T-cell clones for each T-cell subtype in CSF (**D**, *n* = 3) and PBMCs (**E**, *n* = 3). Each dot represents an individual sample’s shared T-cell clone proportion. (**F**) Bar plot showing the proportion of shared and unshared T-cell clones according to clonal size in CSF (*n* = 3) and PBMCs (*n* = 3). (**G**) Heatmap showing DEGs in CD8^+^ TEM cells between CSF, PBMC-shared, and PBMC-unshared T-cell clones. TCM, central memory T cell.

To elucidate the connection between CSF and PBMC T cells, we conducted additional investigations on single-cell RNA and TCR sequencing of matched CSF–PBMC samples from three patients with sporadic ALS (Fig. 6A and B). The CSF showed a tendency towards lower proportions of both CD4^+^ and CD8^+^ naïve T-cell populations than observed in PBMCs (Supplementary Fig. 6A). The proportions of T-cell clones shared between CSF and PBMCs were 4.6 and 3.6%, respectively. Within these shared clones, CD8^+^  *GZMK*^hi^ TEMs constituted the most prevalent population, with 16.7% of CD8^+^  *GZMK*^hi^ TEMs in the CSF sharing clones with PBMCs (Fig. 6C–E). Furthermore, shared T cells exhibited a larger clonal size than non-shared T cells (Fig. 6F). Subsequently, we evaluated the differences in gene expression profiles between shared and non-shared clones within the CD8^+^ TEM population of PBMCs (Fig. 6G). Among the genes that demonstrated significant differences (*P* < 0.01) between these two clone types, we observed elevated expression of *GZMK* and diminished expression of *GZMB* and *GNLY* in the shared clones (Fig. 6G). Comparisons of gene expression profiles between CSF and PBMC CD8^+^ TEMs also showed elevated expression of *GZMK* in the CSF, while PBMCs exhibited increased expression of *GZMB* and *GNLY* (Supplementary Fig. 6B). Collectively, this evidence suggests that shared CD8^+^ TEM clones within PBMCs display properties closely resembling those found in the CSF.

## Discussion

The role of neuroinflammation in neurodegeneration has been widely recognized,^30^ but relatively less attention has been paid to CD8^+^ T cells than to glial cells or CD4^+^ T cells.^31-33^ In this study, we demonstrated an increase in CD8^+^ T cells that highly expressed *GZMK* in the CSF of patients with sporadic ALS. This cell type showed substantial clonal expansion, which was notably higher in patients with ALS than in controls. As the disease progressed, the proportion of CD4^+^ T cells decreased significantly, while the proportion of CD8^+^ GZMK^hi^ T cells showed an increasing trend. In addition, we found higher expression of chemokines in the CSF macrophages of the ALS group than in those of the control group, suggesting a potential link to the chemotactic recruitment and activation of cytotoxic T cells mediated by macrophages. We also identified the presence of CNS-infiltrating T cells with CD8^+^ TEMRA signatures and observed the co-localization of CD8^+^ T cells with neurons or astrocytes. Lastly, most of the shared clones between the CSF and PBMCs were CD8^+^  *GZMK*^hi^ TEMs, exhibiting similar properties in both compartments.

The composition of T cells differed between individuals with ALS and controls. Under normal conditions, ∼70% of CSF cells comprise CD4^+^ T cells.^34^ In accordance with this, CD4^+^ T cells accounted for 61% of CSF cells in the control group in our study, but this proportion was lower (45.2%) in the ALS group. Studies have shown that other neurodegenerative diseases are linked to a reduction in CD4^+^ T cells in PBMCs.^35,36^ A relationship was also observed between a lower proportion of CD4^+^ T cells and greater clinical severity in patients with ALS. The most significant alteration in the CSF T-cell subset of patients with ALS was the increase in CD8^+^  *GZMK*^hi^ TEMs. While a recent study revealed no difference in the proportion of CD8^+^ T cells in the CSF of patients with sporadic ALS versus the control group,^37^ it is noteworthy that this study categorized CD8^+^ T cells as either activated or non-activated. The CD8^+^  *GZMK*^hi^ TEMs in our study showed high expression of *TIGIT* and *TOX* and low expression of *CCR7* and *TCF7*, which are hallmarks of CD8^+^ TEMRAs.^13^  *TIGIT*, which encodes a co-inhibitory receptor on T cells,^38^ and *TOX*, a transcriptional regulator associated with immune cell exhaustion,^39^ are known to exhibit increased transcription upon chronic antigen stimulation.^40^ Nevertheless, high *TOX* expression does not necessarily signify hypofunctional T-cell exhaustion,^41^ as recent research indicates the role of *TOX* in maintaining self-reactive T cells.^42^ Moreover, *ENTPD1*, the hallmark of exhausted T cells,^43^ was minimally expressed, suggesting that this cell population does not contain substantial amounts of exhausted or dysfunctional T cells. It is plausible that persistent neuronal antigen stimulation leads to an increase in CD8^+^  *GZMK*^hi^ TEMs, potentially contributing to the observed neuroinflammation in ALS. Moreover, we confirmed an increase in CD8^+^ T-cell infiltration within the perivascular spaces and parenchyma of the spinal cord and motor cortices, coupled with the presence of CD8^+^ TEMRAs.

The abundance of CD8^+^  *GZMK*^hi^ TEMs in patients with ALS was not merely a proportional increase. TCR analysis revealed a clear clonal expansion of CD8^+^ T cells, including CD8^+^  *GZMK*^hi^ TEMs, in the ALS group. Continuous stimulation by a specific antigen often leads to the progressive differentiation and clonal expansion of T cells. Hence, chronic neuronal antigen stimulation through the TCR is suggested as the cause of the increased clonal expansion of CD8^+^  *GZMK*^hi^ TEMs in ALS. This aligns with recent studies illustrating the clonal expansion of CD8^+^ T cells in the CSF and PBMCs in neurodegenerative diseases, including Alzheimer’s disease and Parkinson’s disease,^35,36^ as well as in PBMCs of individuals with familial ALS.^13^ These findings collectively imply that an enhanced intrathecal adaptive immune response is a shared characteristic of neurodegenerative disorders.

CNS-specific macrophages consist of microglia and BAMs.^44^ BAMs, as non-parenchymal tissue-resident macrophages, share a common origin with microglia, originating from the yolk sac. Our data revealed that CSF macrophages in patients with ALS expressed not only classic macrophage markers, such as *CSF1R* and *C1QC,* but also BAM markers, such as *LYVE1.*^44^ However, they also expressed microglia-specific markers, such as *TMEM119* and *P2RY12*, in non-negligible proportions.^44^ Taken together, these CSF macrophages might represent a potential population of CSF-floating BAMs or microglia that have migrated from the parenchyma into the CSF. In this study, CSF macrophages from patients with ALS demonstrated significantly increased expression of genes encoding chemotactic substances for cytotoxic T cells, including *CCL3*, *CCL4*, and *CXCL8*. Interestingly, the receptors for these chemokines were specifically expressed particularly by CD8^+^ TEMs in the CSF. This suggests that CSF macrophages in patients with ALS may contribute to the recruitment and activation of CD8^+^ TEMs and, consequently, potentially play a role in the pathogenesis of the condition.

T cells can circulate between peripheral blood and CSF to a limited extent, even though the CSF is largely isolated from the peripheral immune system.^45^ Our TCR analysis with paired CSF and PBMC samples enabled us to identify T-cell clones present in both compartments, primarily within the CD8^+^  *GZMK*^hi^ TEM subset. It is highly likely that these T cells encountered an antigen from the CNS, such as peptides from dying motor neurons, and spilled into the peripheral circulation. The fact that the shared T-cell clones in PBMC exhibited a CSF-like profile provides additional evidence that this population represents a CSF–blood-patrolling T-cell subset. The most notable gene expression profiles of these shared clones were high *GZMK* and low *GZMB*. Both *GZMK* and *GZMB* are genes that are translated into cytotoxic molecules, granzyme K and granzyme B, respectively. Interpretations related to this observation can be derived from research on other inflammatory diseases. In the synovial fluid of inflamed joints, *GZMK*^hi^ CD8^+^ T cells and *GZMB*^hi^ CD8^+^ T cells have been found to exhibit distinct features.^46^  *GZMK*^hi^ CD8^+^ T cells were abundant in inflamed synovial tissue and also circulated; furthermore, their gene expression profile was similar to that of the CSF–PBMC-shared clones in our data. These results suggest that inflamed tissue-specific CD8^+^ T cells highly expressing *GZMK* also patrol the peripheral circulation in patients with neurodegenerative disorders, similar to their role in inflammatory diseases.

Our study has several strengths, including the presentation of CNS-derived CD8^+^ T-cell signatures obtained from paired CSF and PBMC samples of patients with evidently sporadic ALS, complemented by their genetic information. Despite the small sample size, our identification of clonally expanded CD8^+^  *GZMK*^hi^ TEMs in the CNS suggests that they could be related to disease progression and exhibit region specificity favouring the motor cortices. Finally, our findings suggest that adaptive immune cells could serve as a potential biomarker to monitor the progression of ALS and that modulating these cytotoxic T cells may offer a novel approach to modifying the clinical course of the disease. Nevertheless, there are several limitations to consider when interpreting the results of this study. First, the small sample size in this study posed challenges in achieving statistical significance and exploring associations with clinical factors. Additionally, while further validation of our key findings through methods such as flow cytometry would have provided additional support for our results, this was not feasible due to the limited availability of CSF specimens. Therefore, a larger-scale study would be required in the future to establish the correlation between the clinical features of ALS and the profile of CD8^+^ T cells in the CSF and to validate these findings using complementary techniques. Second, the control group was not age-matched to the ALS group. Due to the difficulty in obtaining CSF samples from non-inflammatory disease controls, we had to use the available CSF samples. To address potential age-related bias, we examined correlations within the ALS group between age and both CD8^+^  *GZMK*^hi^ TEMs proportion and the clonality of CD8^+^ T cells, finding no significant relationships. Additionally, our elderly control subject (CTRL2, 60+ years) did not show elevated CD8^+^  *GZMK*^hi^ TEMs proportion compared with younger controls. Nevertheless, since age is a major factor influencing immune cell state, caution is needed when interpreting the results. Third, the patients with ALS included in the single-cell RNA sequencing analysis were in the early to middle stages of the disease, whereas brain autopsy samples corresponded to the terminal stage. However, the findings suggest that neuroinflammation plays a crucial role in ALS from the onset of symptoms extending to the terminal stage.

Overall, we demonstrated an increased adaptive immune response in the CNS of patients with ALS, particularly associated with cytotoxic T cells. We observed an increased proportion and clonality of CD8^+^  *GZMK*^hi^ T cells in CSF and demonstrated their communication with the peripheral blood. Furthermore, activated CSF macrophages may play a role in the recruitment and activation of T cells. Collectively, these findings suggest that CD8^+^  *GZMK*^hi^ T cells, exposed to persistent antigen and TCR stimulation in the CNS, contribute to the pathogenesis of ALS.

## Supplementary Material

fcae428_Supplementary_Data

## Data Availability

The processed or raw single-cell RNA and TCR data are available from the corresponding authors upon reasonable request. The Python code used for the analysis is available at https://github.com/arimthebig/ALS.
