# Supplementary material for: Single-cell analysis reveals expanded CD8+ GZMKhigh T cells in CSF and shared peripheral clones in sporadic amyotrophic lateral sclerosis
Source: Brain Commun. 2024 Nov 27;6(6):fcae428. doi: 10.1093/braincomms/fcae428 (PMC11631212; doi:10.1093/braincomms/fcae428)
Supplement: fcae428_Supplementary_Data [file fcae428_supplementary_data.pdf]

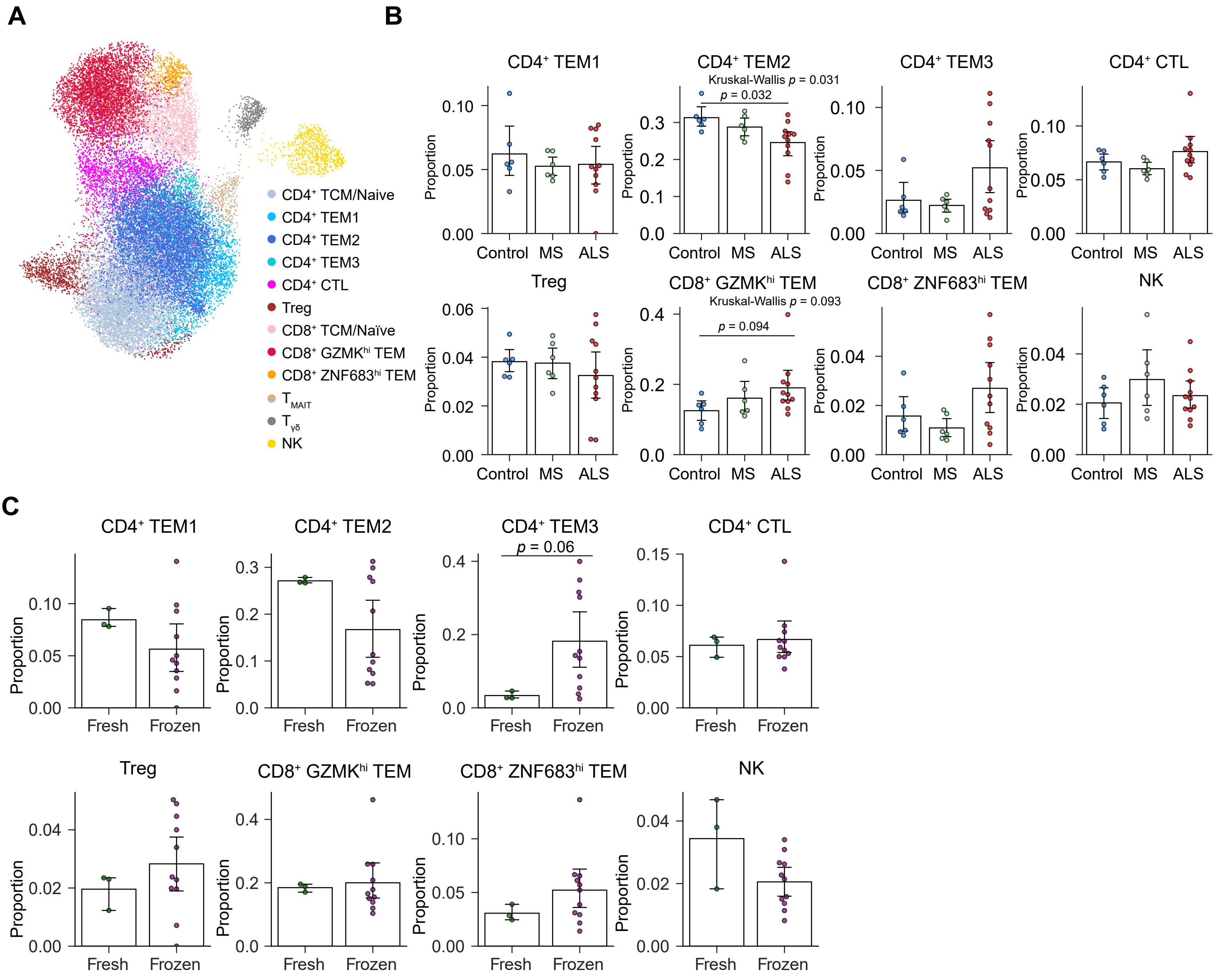

Supplementary Figure 1. T and NK cell subset proportions: comparison with public data and the impact of fresh vs. frozen sample

(A) UMAP of T and NK cell groups combining GSE133028 data and our private data. (B) Bar plots comparing T cell subset proportions, including public data, among control (N = 6), multiple sclerosis (MS, N = 6), and amyotrophic lateral sclerosis (ALS, N = 11) patients. P-values were calculated using the Kruskal-Wallis test. (C) Bar plots comparing T cell subset proportions within the ALS group based on whether the samples were sequenced after being frozen and thawed (N = 8) or in a fresh state (N = 3) prior to sequencing. P-values were calculated using the Mann–Whitney U test.

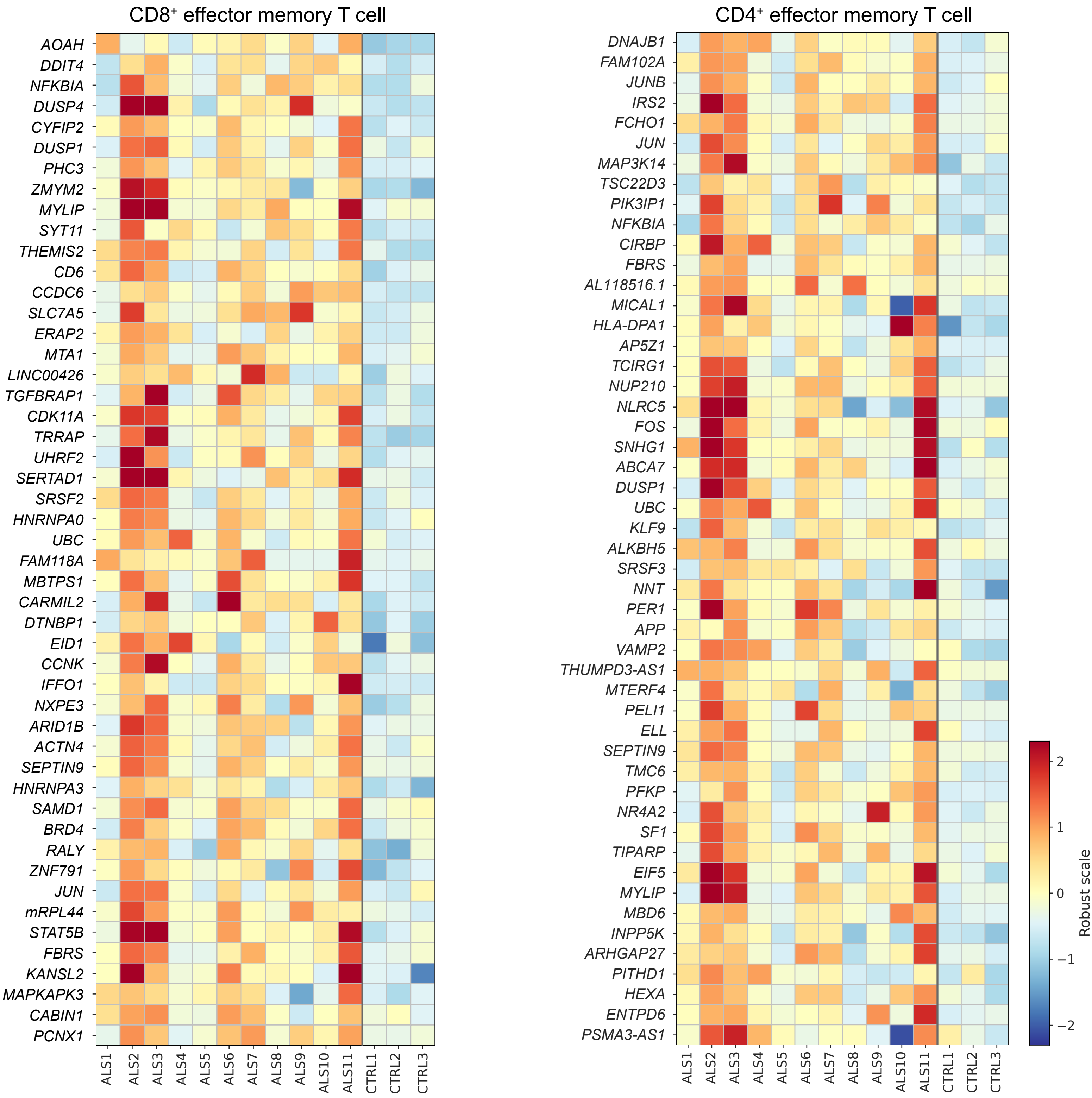

Supplementary Figure 2. Differentially expressed genes of CD8+ and CD4+ TEMs in the CSF
Heatmap of differentially upregulated genes of CD8+ and CD4+ TEMs in ALS patients (N = 11) and controls (N = 3). The genes shown in these heatmaps include differentially upregulated genes ($p < 0.05$) identified through a generalized linear model implemented in pyDESeq2.

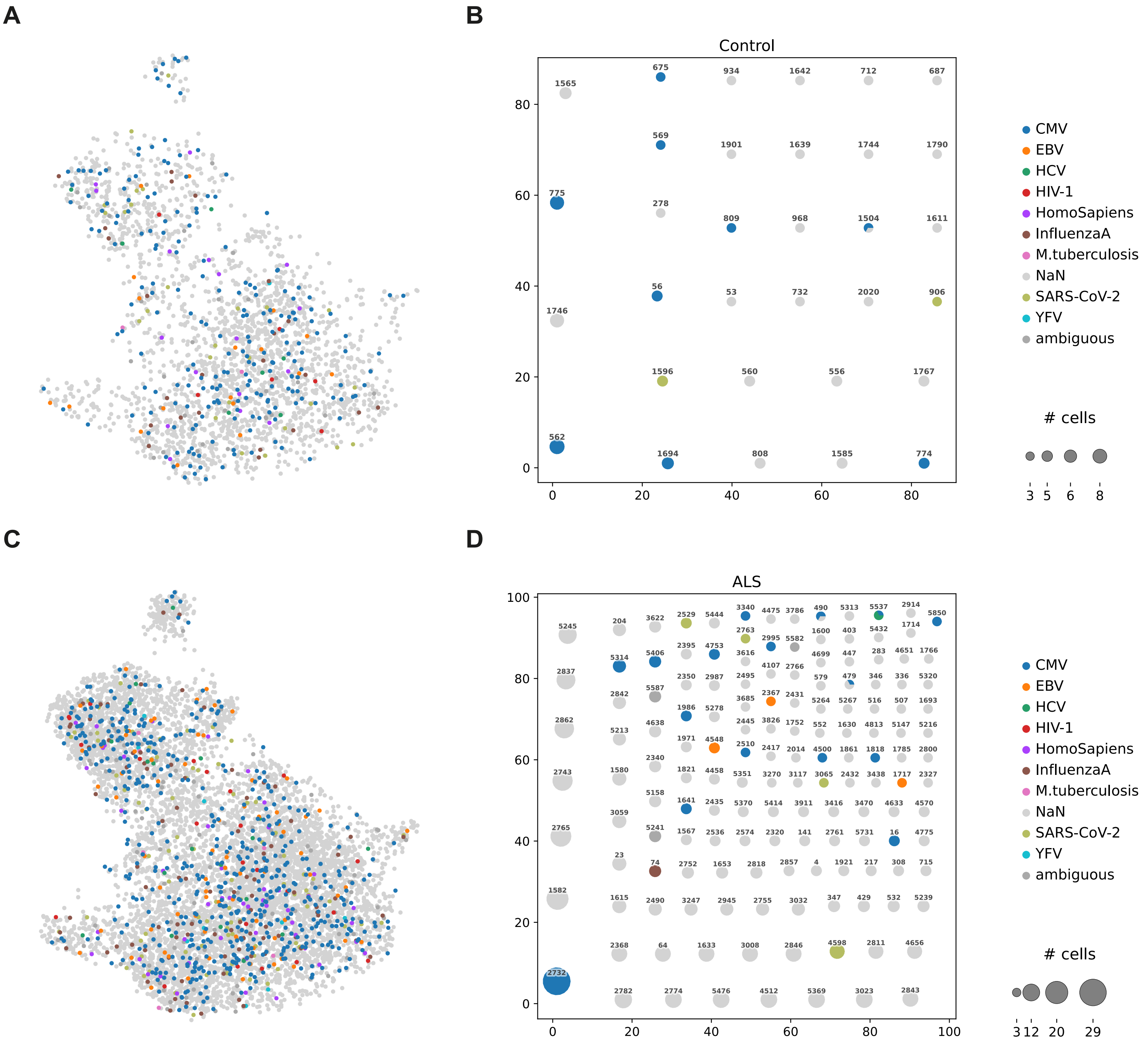

Supplementary Figure 3. Predictive antigen-specific motif of T cell receptor

(A) UMAP visualization of CSF T cells from non-inflammatory disease controls, colored by the antigen-specific motif. (B) Visualization of clone number and clonal size, demonstrating their relationship to the antigen-specific motif in controls. (C) UMAP visualization of CSF T cells from ALS patients, colored by the antigen-specific motif. (D) Visualization of clone number and clonal size, demonstrating their relationship to the antigen-specific motif in ALS patients.

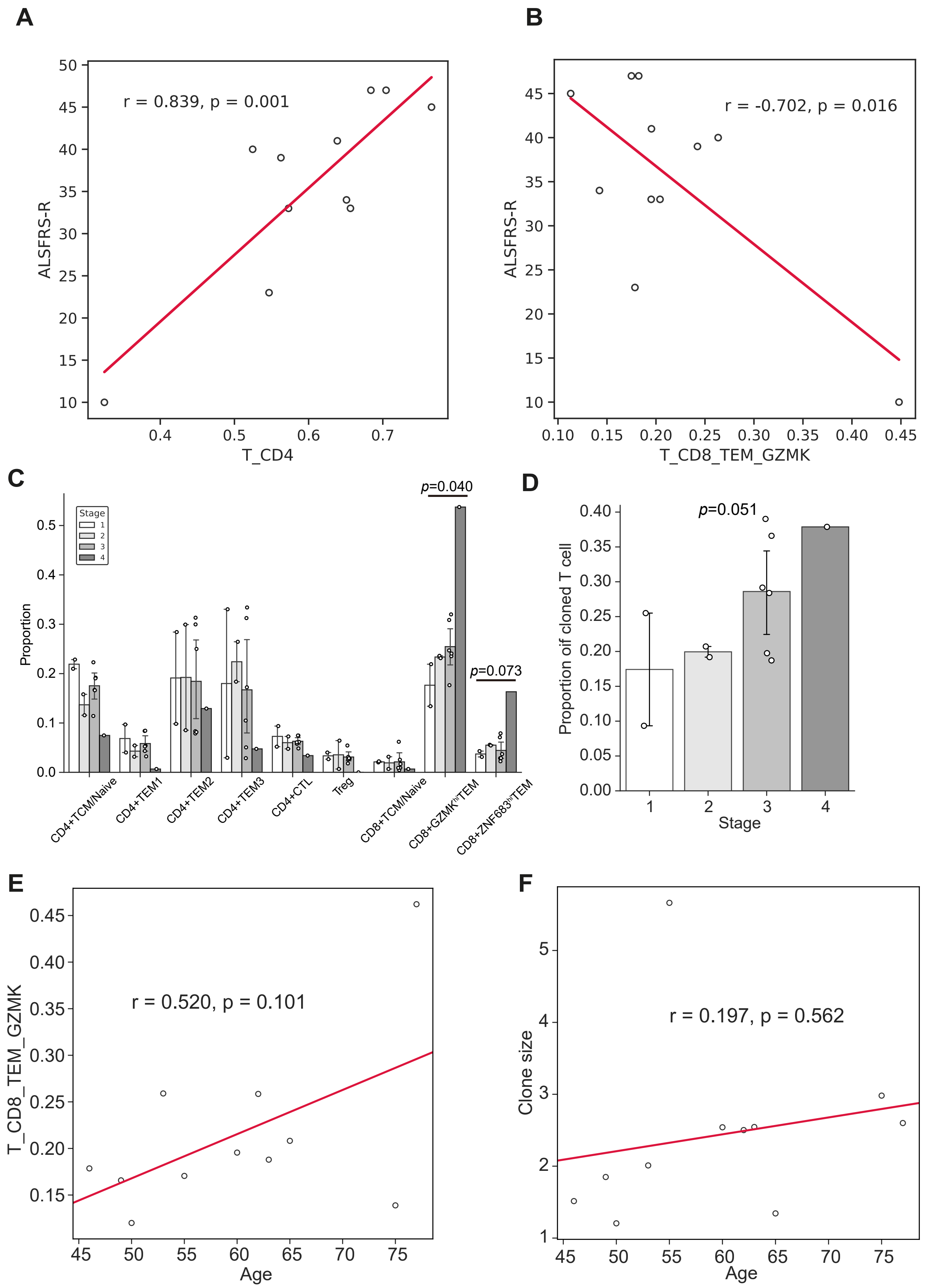

Supplementary Figure 4. Associations between T cell properties and clinical severity or age

(A) Correlation between the proportion of CD4+ naïve or central memory T cells and Amyotrophic Lateral Sclerosis Functional Rating Scale-Revised (ALSFRS-R) scores. Pearson correlation coefficients were calculated to assess the linear relationship. Correlation coefficients (r values) and corresponding p-values are displayed. (B) Correlation between the proportion of CD8+ GZMKhi TEM cells and ALSFRS-R scores. Pearson correlation coefficients were calculated to assess the linear relationship. (C) Bar plot showing the relationship between the proportion of each T cell subtype and ALS clinical stage (N = 11; Stage 1: n = 2, Stage 2: n = 2, Stage 3: n = 6, Stage 4: n = 1). The bars indicate the mean proportion for each group, and error bars represent the standard error of the mean. Proportions were analyzed using the ordinal logistic regression. CD8+ GZMKhi TEMs was significantly increased with advancing stages (p=0.04). (D) Bar plot depicting the relationship between the proportion of clonal CD8+ T cells and ALS clinical stage. The bars indicate the mean proportion for each group, and error bars represent the standard error of the mean. Proportions were analyzed using the ordinal logistic regression. An increasing trend was observed across stages (p=0.051) (E) Correlation between the proportion of CD8+ GZMKhi TEMs and age within ALS group. Pearson correlation coefficients were calculated to assess the linear relationship. (F) Correlation between the mean clone size of CD8+ T cells and age within ALS group. Pearson correlation coefficients were calculated to assess the linear relationship.

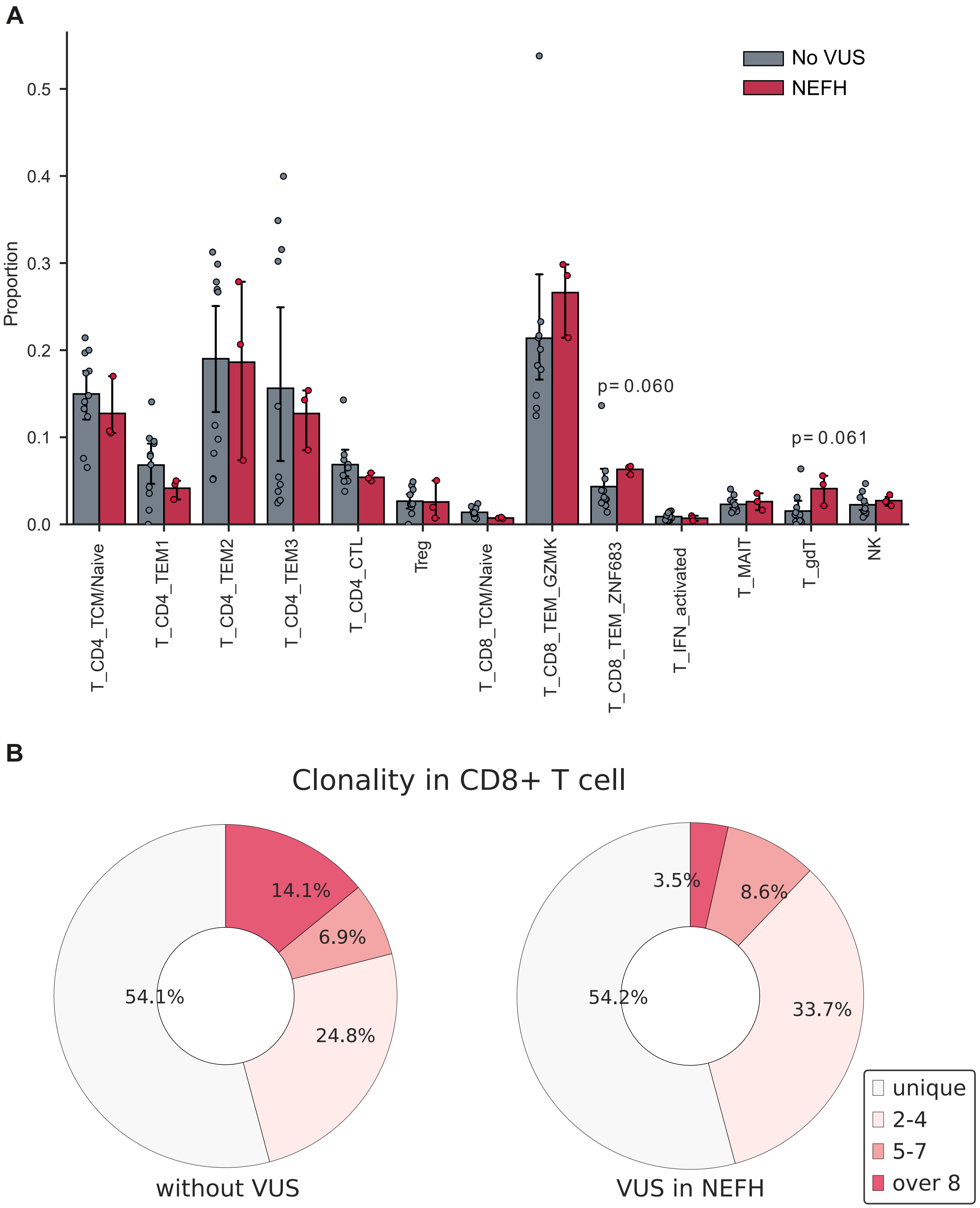

Supplementary Figure 5. Comparisons of T cell properties according to the *NEFH* variants in ALS patients

(A) Bar plot illustrating the proportion of each T cell subtype according to the presence of a variant of uncertain significance (VUS) in the *NEFH* gene (Patient without a VUS, N = 8; those with a VUS in the *NEFH* gene, N = 3). The bars indicate the mean proportion for each group, and error bars represent the standard error of the mean. P-values were calculated using the Mann–Whitney U test. $CD8^+$ *ZNF683*^{hi} TEM and gamma delta T cell populations tended to be higher in ALS patients harboring *NEFH* variants compared to those without, with p-values of 0.060 and 0.061, respectively. (B) Pie chart showing the proportions of clonally expanded CD8+ T cell populations in ALS patients without a VUS (N = 8) versus those with a VUS in the *NEFH* gene (N = 3).

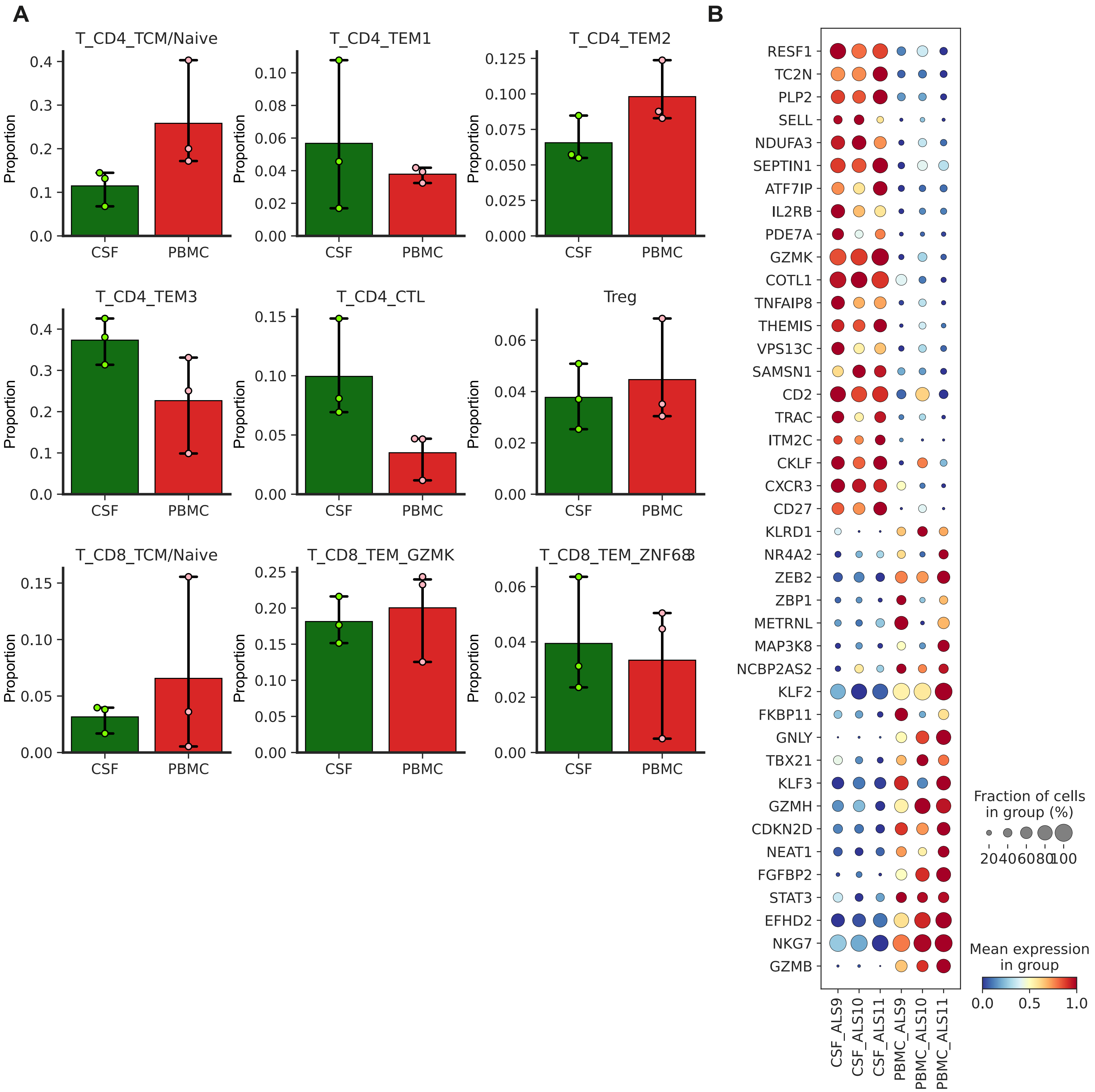

Supplementary Figure 6. Comparisons of the proportions of T cell subtypes and gene expression profiles of CD8+ TEMs between CSF and PBMCs

(A) Bar plot showing the proportion of each T cell subset in CSF (N = 3) and PBMCs (N = 3). Each dot represents an individual sample's proportion within the respective group. The bars indicate the mean proportion for each group, and error bars represent the standard error of the mean. P-values were calculated using the Mann–Whitney U test. (B) Dot plot depicting differential gene expression profiles between CSF and PBMC CD8+ TEMs. Differentially expressed genes were calculated using the Wilcoxon method implemented in Scanpy.

Supplemental Table 1. Clinical Characteristics of the Study Participants (ALS 11 vs. Control 3)

No.	Sample	Sex/Age	Onset	Duration	ALSFRS-R	Stage	DPR	Gene	CSF lab	CSF amount	Multiplexing
ALS1	CSF	M/54	C	9	47	1	0.111	None	R0 W0	15 ml	
ALS2	CSF	M/59	C	13	41	3	0.538	None	R0 W2	15 ml	
ALS3	CSF	F/63	LS	13	33	3	1.153	None	R3 W0	15 ml	
ALS4	CSF	F/50	C	20	45	1	0.150	None	R0 W0	10 ml	3-plex
ALS5	CSF	M/61	LS	25	40	3	0.320	*NEFH* (VUS)*	R0 W4	10 ml	3-plex
ALS6	CSF	M/53	C	26	39	3	0.346	*NEFH* (VUS)†	R0 W1	15 ml	3-plex
ALS7	CSF	M/46	LS	6	47	2	0.166	*NEFH* (VUS)‡	R0 W2	10 ml	3-plex
ALS8	CSF	F/76	B	3	10	4a	12.66	None‡	R0 W0	10 ml	3-plex
ALS9	CSF/PBMC	F/64	C	8	33	2	1.875	ND	R0 W0	10 ml	3-plex
ALS10	CSF/PBMC	M/48	C	10	34	3	1.400	None	R0 W0	10 ml	3-plex
ALS11	CSF/PBMC	F/74	B	6	23	3	4.166	*SQSTM1* (VUS)<br>*ATXN2* (VUS)<br>*TAF15* (VUS)	R0 W0	10 ml	3-plex
Cont1	CSF	M/24	Pseudoseizure						R0 W1	15 ml	
Cont2	CSF	F/63	Ischemic trochlear nerve palsy						R0 W2	15 ml	
Cont3	CSF	M/22	Compressive radial neuropathy						R0 W0	10 ml	

Abbreviations: ALSFRS-R, amyotrophic lateral sclerosis functional rating scale-revised; DPR, disease progression rate; ND, not done; Stage, King's clinical stage; VUS, variant of uncertain significance.

Disease progression rate was calculated as follows: (48 – ALSFRS-R) / (time from onset to assessment [months]).

The first version of ALS gene panel (27 genes) includes *ALS2, ANG, ATXN2, C9orf72, CHCHD10, CHMP2B, ERBB4, FIG4, FUS, GLE1, HNRNPA1, MATR3, NEFH, OPTN, PFN1, SETX, SIGMAR1, SOD1, SPG11, SPG20, SQSTM1, TAF15, TARDBP, TBK1, UBQLN2, VAPB,* and *VCP.*

* Charcot-Marie-Tooth gene panel (73 genes) includes *AARS, ABHD12, AIFM1, ARHGEF10, ATP1A1, ATP7A, BAG3, BSCL2, CNTNAP1, COA7, DCTN1, DCTN2, DGAT2, DHTKD1, DNAJB2, DNM2, DNMT1, DRP2, DYNC1H1, EGR2, FGD4, FIG4, GARS, GDAP1, GJB1, GNB4, HARS, HINT1, HSPB1, HSPB3, HSPB8, IGHMBP2, INF2, KIF1B, KIF5A, LITAF, LMNA, LRSAM1, MARS, MCM3AP, MED25, MFN2, MME, MORC2, MPV17, MPZ, MTMR2, NAGLU,*

NDRG1, NEFH, NEFL, PDK3, PLEKHG5, PMP2, PMP22, PRPS1, PRX, PTRH2, RAB7A, SBF1, SBF2, SCO2, SETX, SGPL1, SH3TC2, SIGMAR1, SPG11, SPTLC1, TRIM2, TRPV4, VCP, WARS, and *YARS. SOD1, TARDBP, FUS, and SETX were* ***not*** *tested.*

† Whole genome sequencing data were analyzed.

‡ The second version of ALS gene panel (48 genes) includes *ALS2, ANG, ANXA11, ATXN1, ATXN2, C9orf72, CAMTA1, CCNF, CFAP410, CHCHD10, CHMP2B, CYLD, CYP27A1, DCTN1, ERBB4, EWSR1, FIG4, FUS, GBA2, GLE1, GRN, HFE, HNRNPA1, HNRNPA2B1, KIF5A, MAPT, MATR3, NEFH, NEK1, NIPA1, OPTN, PFN1, PRPH, SETX, SIGMAR1, SOD1, SPART, SPAST, SPG11, SQSTM1, TAF15, TARDBP, TBK1, TUBA4A, UBQLN2, VAPB, VCP,* and *VRK1.*

Supplemental Table 2. List of Autopsies (ALS 3 vs. Control 4)

No.	Sex/Age	Pathological diagnosis	Other diagnosis	*APOE* genotype
A18-07	M/72	FTLD-ALS (TDP-43)	NA	ε3/ε3
A19-17	F/63	FTLD-ALS (TDP-43)	NA	ε2/ε3
A22-01	F/56	FTLD-ALS (TDP-43) & PART	NA	ε3/ε3
A17-15	F/74	PART	Pneumonia	ND
A18-10	M/56	PART	Hypopharyngeal cancer	ε3/ε3
A22-08	M/61	PART	Lung cancer, M/parietal lobe	ND
A22-20	F/77	PART	Pancreatic cancer	ε3/ε3

Abbreviations: FTLD-ALS, frontotemporal lobar degeneration-amyotrophic lateral sclerosis; NA, not applicable; ND, not done; PART, primary age-related tauopathy.
